# Natural compounds of medicinal plants as a source for novel anti-orthopoxvirus medications

**DOI:** 10.1093/lifemedi/lnag009

**Published:** 2026-04-09

**Authors:** Eman A Makhlouf, Riham A El-Shiekh, Mona M Okba, Hossam M Ashour

**Affiliations:** Clinical Pharmacy Program, Faculty of Pharmacy, Cairo University, Cairo 11562, Egypt; Department of Pharmacognosy, Faculty of Pharmacy, Cairo University, Cairo 11562, Egypt; Department of Pharmacognosy, Faculty of Pharmacy, Cairo University, Cairo 11562, Egypt; Department of Integrative Biology, College of Arts and Sciences, University of South Florida, St. Petersburg, FL 33701, United States

Natural products are increasingly recognized as promising sources of antiviral agents against Mpox. Current *in silico* and *in vitro* findings point to several plant-derived compounds capable of targeting essential viral proteins and limiting Mpox virus (MPXV) replication.

The global resurgence of Mpox has intensified efforts to identify antiviral strategies that are novel, safe, affordable, and widely accessible. While several synthetic antivirals, such as tecovirimat, cidofovir, and brincidofovir, are available, their limitations, including toxicity, the risk of resistance, and the high cost of production, have motivated researchers to explore alternative approaches. One promising avenue is the use of natural products. Plants and their secondary metabolites have long been recognized for their antiviral properties, and recent computational and laboratory studies suggest that many of these compounds may interfere with key stages of MPXV life cycle. This research highlights how plant-derived molecules may serve as early-stage candidates for Mpox therapy.

Mpox, a zoonotic disease caused by MPXV, historically circulated mainly in Central and West Africa. However, the 2022 multi-country outbreak marked a substantial shift in its global reach, prompting the World Health Organization (WHO) to declare a Public Health Emergency of International Concern. Multiple factors, such as declining immunity following the end of smallpox vaccination campaigns and increased human–animal interaction, have contributed to this expanded spread. As cases continue to rise and Mpox persists outside endemic areas, the need for innovative antiviral therapies is increasingly apparent.

Within this context, natural compounds have drawn attention for their chemical diversity and biological activity. Computational screening has become particularly useful in identifying molecules that could interfere with essential viral functions. Among the viral targets under investigation are the VP39 methyltransferase and DNA topoisomerase [1, 2], the envelope protein F13, DNA-dependent RNA polymerase (DdRp), and the F3L protein, which play a role in immune evasion. Disrupting the activity of these proteins can limit viral replication and impair viral spread, which can complement existing host antiviral responses.

MPXV DdRp, a key enzyme in viral transcription, has also been a significant target. Compounds such as salpichrolide J and anabsinthin demonstrated strong and stable binding to the active site of DdRp, outperforming physiological nucleotides in docking analyses [3]. Similarly, punicalagin, an ellagitannin found in pomegranate, showed higher affinity for the E8 protein than several commercially available compounds and maintained stability in simulation studies. This recurrent pattern across different compound classes highlights the versatility of natural products in engaging multiple viral targets.

Beyond computational findings, several *in vitro* studies provide biological support for the antiviral potential of natural compounds. Resveratrol, an abundant plant stilbenoid, has demonstrated significant inhibition of vaccinia virus (VACV) replication and has also been shown to reduce MPXV replication. Resveratrol had minimal impact on early gene expression but strongly suppressed viral DNA synthesis and later-stage gene expression. These findings suggest that resveratrol interferes with replication processes that occur after initial viral entry [4].

When looking collectively at the available findings, several themes emerge. Polyphenols, including curcumin, myricetin, ellagic acid, and resveratrol, repeatedly appear as strong inhibitors of key Mpox viral proteins. Triterpenoids such as betulinic and ursolic acids, and the amyrins, similarly, demonstrate compelling antiviral potential. Some compounds with historical or nutritional relevance, such as folic acid and punicalagin, also show structural compatibility with essential viral targets. This broad distribution of active molecules across different chemical classes speaks to the rich antiviral landscape of natural products. In Fig. 1, some natural products with potential antiviral activities against Mpox are highlighted, including their sources and mechanisms of action. This summarized illustration emphasizes promising natural agents that can be beneficial for further research.

**Figure 1.**
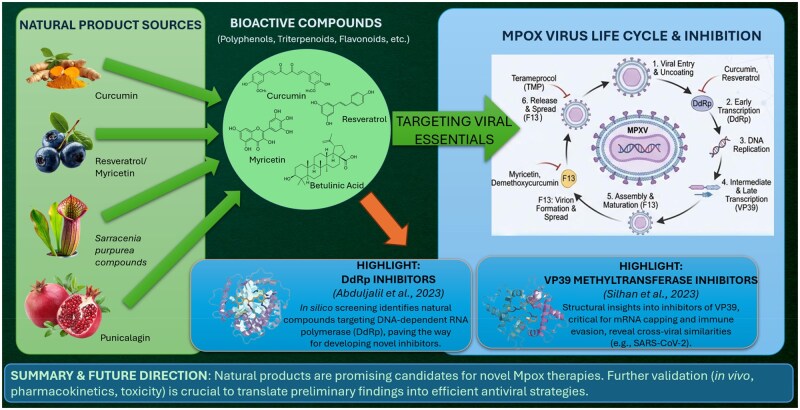
Natural products as promising antiviral agents against Mpox.

Nevertheless, important limitations remain. Many promising computational results have yet to be validated *in vitro* or *in vivo*, and the majority of *in vitro* studies so far have used surrogate viruses rather than MPXV itself. Data on cytotoxicity, pharmacokinetics, and interactions between compounds are also limited. However, the diversity of active natural molecules identified to date suggests considerable potential for developing new antiviral agents from plant sources.

Ultimately, natural compounds provide a versatile and underexplored collection of chemical structures capable of targeting multiple aspects of MPXV biology. Their capacity to bind viral proteins involved in replication, immune evasion, and virion maturation highlights their relevance in the search for new therapeutic strategies. While further studies are needed to confirm the activity of these compounds and refine their pharmacological profiles, the current body of evidence offers a strong rationale for continuing to investigate natural products as a source of future Mpox antivirals.

We would like to highlight two main papers. The first is a paper by Abduljalil et al. that directly targets the core replication enzyme (DdRp) with *in silico* analysis of natural compounds, reflecting a cutting-edge antiviral strategy against MPXV and framing natural products as a credible source for inhibitors [3]. The recent study is broad in scope and is tightly tied to a key viral function.

The study conducts *in silico* screening of natural product libraries against the Mpox virus DdRp, a pivotal enzyme for viral replication. The authors identify several natural compounds with favorable predicted binding affinities and plausible interactions with the catalytic and substrate-binding regions of DdRp. This work illustrates the feasibility of repurposing or sourcing natural products as potential inhibitors and highlights specific chemical scaffolds that merit experimental validation. The emphasis on DdRp as a drug target aligns with broader antiviral strategies that seek to intercept viral replication at its core.

Targeting Mpox replication via DdRp with natural product inhibitors is a promising, early-stage approach that bridges natural product chemistry with antiviral target biology. The study provides candidate scaffolds and a roadmap for rapid experimental follow-up, underscoring the ongoing exploration of alternative chemical spaces in Mpox therapeutics.

There are areas of opportunity for further research, as the conclusions of the paper are inherently computational and rely on docking scores, binding energy estimates, and static protein structures. There needs to be more consideration of enzyme dynamics, cellular uptake, pharmacokinetics, and potential off-target effects. This would ensure that any findings related to the predicted inhibitors can translate into real-world antiviral activity. There is also a need for more experimental validation through *in vitro* enzymatic assays with Mpox DdRp, followed by antiviral cell-based assays to assess potency and cytotoxicity. Structure–activity relationship (SAR) studies around the highlighted scaffolds could refine binding and selectivity. Pharmacokinetic profiling and toxicity screening would be important prior to any translational work. Finally, expanding the *in silico* pipeline to include molecular dynamics and free-energy perturbation calculations could improve predictive power and help prioritize candidates for synthesis or procurement.

The second study, authored by Silhan et al. [5], shows that VP39 (methyltransferase) is a critical enzyme for viral mRNA capping and immune evasion. This work provides structural insights and identifies inhibitors, representing state-of-the-art drug-design approach with direct mechanistic relevance to current MPXV biology and direct relevance to SARS-CoV-2 research.

The article reports the discovery and structural characterization of inhibitors targeting Mpox virus VP39, a methyltransferase essential for cap formation on viral mRNA and immune evasion. The authors provide high-resolution structural data on inhibitor binding modes and reveal notable structural similarities between VP39 and SARS-CoV-2 nsp14 methyltransferases, suggesting conserved pharmacophores across orthogonal viruses. The work advances our understanding of the mechanics of Mpox mRNA capping and presents a credible path for rational design of VP39 inhibitors.

Structural inhibition of Mpox VP39 methyltransferase represents a targeted approach to disrupt viral cap formation and immune evasion, with compelling cross-links to SARS-CoV-2 research. This work provides a concrete structural framework for developing selective VP39 inhibitors and can guide future medicinal chemistry leaps in this area of research.

The findings are based on biophysical and structural analyses, with inhibitor binding demonstrated in purified systems. There is limited information on cellular activity, antiviral efficacy in cell culture, or *in vivo* relevance. Potential issues include selectivity against host methyltransferases and the pharmacological properties of the inhibitors. The cross-viral comparison, while intriguing, may require broader validation across related enzymes and viral contexts.

Further work should include cellular antiviral assays to evaluate efficacy and cytotoxicity, selectivity profiling against host methyltransferases, and optimization of inhibitors for cell permeability and metabolic stability. Additional structural studies with diverse VP39 variants and related enzymes could map resistance surfaces. Cross-validation with SARS-CoV-2 nsp14 inhibitors could inform broader antiviral design strategies and potential pan-viral methyltransferase inhibitors.

Finally, a general limitation is the extensive nature of Mpox virus variability and possible resistance mutations, which studies may not fully account for.

## References

[1] Manikyam HK , Patil SB, Hussain N et al High-throughput *insilico* drug screen against mpox targeted proteins in comparison with repurposed antiviral drugs against natural compounds. J Pharm Res Int 2024;36:41–52.

[2] Adepoju OA , Danazumi AU, Dibba LBS et al Computational interrogation of natural compounds identified resveratrol-3-O-D-glucopyranoside as a potential inhibitor of essential monkeypox virus proteins. Intell Med 2025;5:5–13.

[3] Abduljalil JM , Elfiky AA, Elgohary AM et al Exploration of natural compounds against the human mpox virus DNA-dependent RNA polymerase in silico. J Infect Public Health 2023;16:996–1003. 37167647 10.1016/j.jiph.2023.04.019 PMC10148721

[4] Cao S , Realegeno S, Pant A et al Suppression of poxvirus replication by resveratrol. Front Microbiol 2017;8:2196. 29204136 10.3389/fmicb.2017.02196 PMC5698801

[5] Silhan J , Klima M, Otava T et al Discovery and structural characterization of monkeypox virus methyltransferase VP39 inhibitors reveal similarities to SARS-CoV-2 nsp14 methyltransferase. Nat Commun 2023;14:2259. 37080993 10.1038/s41467-023-38019-1 PMC10116469

